# Comparison of the long-term clinical performance of a biodegradable and a titanium fixation system in maxillofacial surgery: A multicenter randomized controlled trial

**DOI:** 10.1371/journal.pone.0177152

**Published:** 2017-05-11

**Authors:** B. Gareb, N. B. van Bakelen, G. J. Buijs, J. Jansma, J. G. A. M. de Visscher, Th. J. M. Hoppenreijs, J. E. Bergsma, B. van Minnen, B. Stegenga, R. R. M. Bos

**Affiliations:** 1 Department of Oral and Maxillofacial Surgery, University Medical Centre Groningen, University of Groningen, Groningen, The Netherlands; 2 Department of Oral and Maxillofacial Surgery, Medical Centre Leeuwarden, Leeuwarden, The Netherlands; 3 Department of Oral and Maxillofacial Surgery, Rijnstate Hospital Arnhem, Arnhem, The Netherlands; 4 Department of Oral and Maxillofacial Surgery, Amphia Hospital Breda, Breda, The Netherlands; 5 UMCG Centre for Dentistry and Oral Hygiene, Department Oral Health Care & Clinical Epidemiology, University Medical Centre Groningen, University of Groningen, Groningen, The Netherlands; UNITED STATES

## Abstract

**Background:**

Biodegradable fixation systems could reduce or eliminate problems associated with titanium removal of implants in a second operation.

**Aim:**

The aim of this study was to compare the long-term (i.e. >5 years postoperatively) clinical performance of a titanium and a biodegradable system in oral and maxillofacial surgery.

**Materials and methods:**

The present multicenter Randomized Controlled Trial (RCT) was performed in four hospitals in the Netherlands. Patients treated with a bilateral sagittal split osteotomy (BSSO) and/or a Le Fort-I osteotomy, and those treated for fractures of the mandible, maxilla, or zygoma were included from December 2006 to July 2009. The patients were randomly assigned to either a titanium (KLS Martin) or a biodegradable group (Inion CPS).

**Results:**

After >5 years postoperatively, plate removal was performed in 22 of the 134 (16.4%) patients treated with titanium and in 23 of the 87 (26.4%) patients treated with the biodegradable system (P = 0.036, hazard ratio (HR) biodegradable (95% CI) = 2.0 (1.05–3.8), HR titanium = 1). Occlusion, VAS pain scores, and MFIQ showed good and (almost) pain free mandibular function in both groups.

**Conclusion:**

In conclusion, the performance of the Inion CPS biodegradable system was inferior compared to the KLS Martin titanium system regarding plate/screws removal in the abovementioned surgical procedures.

**Trial registration:**

http://controlled-trials.com ISRCTN44212338.

## Introduction

Titanium osteosynthesis is currently the fixation system of choice in maxillofacial traumatology and orthognathic surgery. According to literature in 5–40% of the cases, titanium osteosynthesis material is removed in a second operation following adequate bone healing because of infections or other clinical symptoms [1–5].

Biodegradable osteofixation systems have the ability to degrade in the human body, which ideally could reduce or even eliminate removal of implants during a second operation [6]. However, adverse tissue reactions against degradation products have been reported [7–9]. Consequently, biodegradable implants are removed in a second operation in 0–31% of the cases [4,10,11].

Most studies in the literature comparing titanium versus biodegradable osteofixation systems lack a control group or have insufficient follow-up [12]. In 2006, we started a randomized controlled trial comparing titanium vs. biodegradable plates and screws in maxillofacial surgery [3]. Short-term (i.e. 8 weeks) healing outcomes were similar in both groups [3]. However, the risk of removal of biodegradable plates and screws was 2.2 times higher compared to titanium implants, within the first 2 postoperative years [4]. Although these results are of importance, studies focusing on long-term outcome (i.e. >5 years) are needed since host response and full degradation and resorption of implants can take up to 4 or 5 years [9,13–16]. Additionally, removal of titanium plates and screws three to five years after surgery has been reported [1,17]. The present study is part of the abovementioned randomized controlled trial [3].

The aim of the present study was to compare the long-term (i.e. >5 years postoperatively) clinical performance (i.e. removal of the plate/screws) of the titanium and the biodegradable system following fixation of mandibular, Le Fort-I, and zygomatic fractures, and bilateral sagittal split osteotomies (BSSO) and/or Le Fort-I osteotomies.

## Material and methods

This Randomized Controlled Trial (RCT) has been described according to the CONSORT statement 2010 (http://www.consort-statement.org/). Trial registration date and number: 28 December 2006, ISRCTN44212338 (http://controlled-trials.com). The authors confirm that all ongoing and related trials for this drug/intervention are registered.

### Study population

The recruitment start date of this RCT was October 2006. Patients were included from December 2006 to July 2009. During this period, 230 patients were treated at four different departments of Oral and Maxillofacial (OMF) Surgery in the Netherlands (University Medical Center Groningen, Rijnstate Hospital Arnhem, Amphia Hospital Breda, and Medical Center Leeuwarden). The in- and exclusion criteria are summarized in Table 1. Participants were recruited by OMF surgeons and were randomly assigned to either the titanium or biodegradable treatment group. Randomization occurred a day before (osteotomies) or immediately prior to the operation (fractures). Randomization sequences were generated by a statistician using a computerized randomization program and randomization was performed using an Interactive Voice Response System (IVRS) with block size 10, which was available 24-hours a day to conceal the randomization sequence until the interventions were assigned. Randomization was stratified by hospitals to ensure that both treatment options were equally divided over the participating hospitals. All patients provided written informed consent prior to enrollment and to publication of the work. The study was approved at 1 May 2006 by the Medical Ethical Committees of the participating hospitals in the Netherlands (University Medical Centre Groningen, Rijnstate Hospital Arnhem, Amphia Hospital Breda, and Medical Centre Leeuwarden).

**Table 1:** Inclusion and exclusion criteria.

*Inclusion criteria*:
- patients scheduled for a Le Fort-I fracture, and/or a solitary or multiple (maximum 2) mandibular fracture(s), and/or a zygoma fracture;
- patients scheduled for a Le Fort-I osteotomy, and/or a Bi-lateral Sagittal Split Osteotomy (BSSO);
- patients (also parents or responsible persons if necessary) who signed the *informed consent* form.
*Exclusion criteria*:
- patients who were younger than 18 years old (trauma), or patients who were younger than 14 years (osteotomies);
- patients presented with heavily comminuted fractures of the facial skeleton;
- patients who experienced compromised bone healing in the past;
- patients who were pregnant;
- patients who could/would not participate in a 1-year follow-up (reasons);
- patients who would not agree with an *at random* assignment to one of the treatment groups, or one of the methods or treatment administered in the study;
- patients who were diagnosed with a psychiatric disorder (diagnosed by a psychiatrist);
- patients who experienced cleft lip and palate surgery in the past;
- patients where fracture reduction and fixation was delayed for more than 7 days (after day of trauma);
- patients of whom the general health and/or medication could affect bone healing, as determined by the oral and maxillofacial surgeon.

### Interventions

Patients were assigned to either a titanium control-group (KLS Martin, Gebrüder Martin GmbH&Co. Tuttlingen, Germany) or to a biodegradable test-group (Inion CPS, Inion Ltd. Tampere, Finland). Prior to surgery, patients were blinded for the used system. All plates and screws were applied according to the instructions of the manufacturers.

Fixation of mandibular osteotomies and fractures was performed using 2.5-mm biodegradable or 2.0-mm titanium plates and screws, while 2.0-mm biodegradable or 1.5-mm titanium plates and screws were used for fixation of zygomatic fractures, Le Fort-I fractures, and Le Fort-I osteotomies. Each participating OMF surgeon performed 2 ‘test-surgeries’ using the biodegradable system to acquire the different application-skills, i.e. pre-tapping the screw holes and pre-heating the plates, and getting used to the different dimensions of the material. These ‘test-surgeries’ were not included in the study. The patients did not receive rigid maxillomandibular fixation, but soft guiding elastics post-operatively, and were instructed to use a soft diet for five weeks. It was agreed that routine removals of asymptomatic plates would not be performed.

### Outcome measures

The most important outcome variable in the present study was the removal of the plate/screws (yes;no) after long-term follow-up (i.e. >5 years postoperatively) after treatment with the biodegradable or the titanium system, taken the time from the moment of implantation to removal into account.

The following other outcome measures were assessed:

1. reasons for plate/screws removal;
2. patient-related (self-evaluation): correct occlusion (yes;no), palpability of plates/screws (yes;no), signs of swelling at follow-up in the operation area (yes;no), pain reported on a Visual Analogue Scale (VAS; ranging 1–100), and mandibular function evaluated by the 17 questions of the Mandibular Function Impairment Questionnaire (MFIQ [18]; ranging 17–85: a higher score means worse function);
3. patients were also asked whether they would choose for surgery again if they had known all the implications of the operation in advance (yes;no).

All patients were contacted by telephone >5 years postoperatively to evaluate the outcome measures. In addition, their (electronic) medical records were evaluated for plate/screws removal. The complete date range of patient inclusion until the final follow-up was December 2006 to June 2016. The outcome measures were recorded on Case-Report-Forms.

### Statistical analysis

Inclusion of the 230 patients was based on power analysis using the outcome measure ‘bone healing after 8 weeks’, which is described in detail elsewhere [3]. All normally distributed variables were presented as means and standard deviations (SD). Mean values of both treatment groups were compared using the independent-samples t-test. Not normally distributed continuous data were presented as medians and ranges, and compared using the Mann-Whitney U test. All nominal or categorical variables were described as frequencies and percentages. Comparison between both groups for these variables was performed using the Fisher’s exact test or Chi-squared test.

The difference in plate survival between the biodegradable and the titanium group was presented using a Kaplan-Meier estimator plot and analyzed by the Logrank test. The intra-operative switches from the biodegradable to the titanium system may have influenced plate removal [19]. Therefore, the hazard ratio of the used treatment systems was calculated using a Cox regression analysis, which was adjusted for ‘intra-operative switches’. The estimated plate removal rate was calculated by dividing the number of events (plate removal) by the total plate in situ time. The total in situ time was calculated by taking the sum of:

1. the in situ time up to removal of plates that were removed during the observation period;
2. the in situ time of plates that were not removed and could be followed for the entire observation period. These patients were censored at the day of medical record evaluation in the survival analysis;
3. the in situ time up to the end of observation of plates that were not removed and where patients did not complete the entire observation period. We also viewed the (electronic) medical records. If the records showed no plate removal, no matter if the patients could be reached by telephone, these patients were also censored at the day of medical record evaluation.

P-values less than 0.05 were considered statistically significant. All analyses were performed in Statistical Package of Social Sciences (SPSS) 22 (IBM SPSS Statistics for Windows, Version 22.0. Armonk, NY: IBM Corp.).

## Results

**Fig 1:**
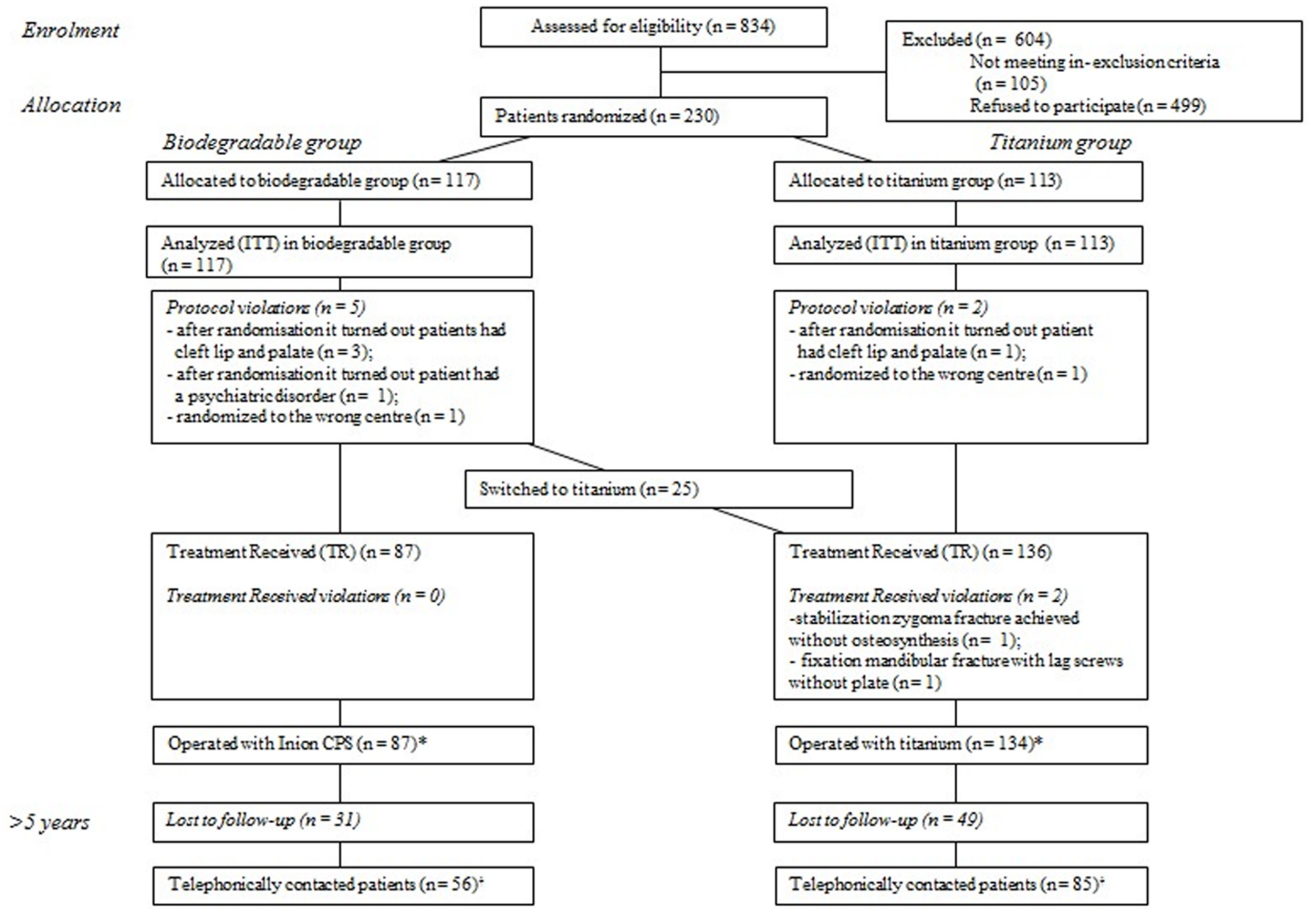
Flow diagram of patient’s progress through the phases of RCT. * Plate removal analyses. ^†^ Analyses of the other variables. ITT = intention-to-treat, n = number.

The flow of the 230 originally included randomized patients is shown in Fig 1. Seven patients (five in the biodegradable and two in the titanium group) were excluded due to protocol violations. In 25 patients who were randomized to the biodegradable group, the OMF surgeon decided to switch to the titanium system intra-operatively [3]. There were 2 titanium treatment received violations. Consequently, the titanium group consisted of 134 patients and the biodegradable group consisted of 87 patients (‘Total included patients’; Table 2). For the long-term follow-up of the present study, 49 (36.5%) and 31 (35.6%) patients were lost to follow-up (LTFU) in the titanium and biodegradable group, respectively. These patients could not be reached by telephone. This resulted in 85 patients in the titanium group and 56 patients in the biodegradable group (‘Contacted patients’; Table 2). There were no significant differences in performed surgical procedures, gender and age distribution, and removal of plates/screws between the LTFU and not LTFU patients (S1 Table).

**Table 2:** Baseline characteristics of the total included and telephonically contacted patients.

Description	Total included patients*			Contacted patients^‡^		
	Titanium (n)	Biodegradable (n)	P-value^†^	Titanium (n)	Biodegradable (n)	P-value^†^
*Surgical procedures*	134	87		85	56	
BSSO	87 (64.9%)	55 (66.3%)	0.795	59 (69.4%)	35 (62.5%)	0.165
Le Fort-I osteotomy	8 (6.0%)	8 (9.2%)		3 (3.5%)	5 (8.9%)	
Bi-maxillary osteotomy	29 (21.6%)	16 (18.4%)		19 (22.4%)	11 (19.6%)	
Mandibular fracture	6 (4.5%)	4 (4.6%)		4 (4.7%)	2 (3.6%)	
Le Fort-I fracture	1 (0.7%)	0		0	0	
Zygoma fracture	3 (2.2%)	4 (4.6%)		0	3 (5.4%)	
*Gender/age distribution*						
Male	55 (41%)	43 (49.4%)	0.268	37 (43.5%)	28 (50%)	0.492
Female	79 (59%)	44 (50.6%)		48 (56.5%)	28 (50%)	
Age (median (range) in years)	29 (16–60)	28 (14–59)	0.786	30 (16–60)	30 (15–59)	0.993

*Analyses performed on all included patients, without the Protocol violations and the Treatment Received violations(see Fig 1), n = 221: titanium n = 134, biodegradable n = 87).

^†^Tested two-tailed.

^‡^ Analyses performed on all telephonically contacted patients after long-term follow-up (i.e. >5 years postoperatively), n = 141: titanium n = 85, biodegradable n = 56).

Abbreviations: BSSO = bilateral-sagittal-split osteotomy, n = number.

All baseline characteristics did not differ significantly between the titanium and biodegradable group in both ‘total included patients’ and ‘contacted patients’ (Table 2).

**Fig 2:**
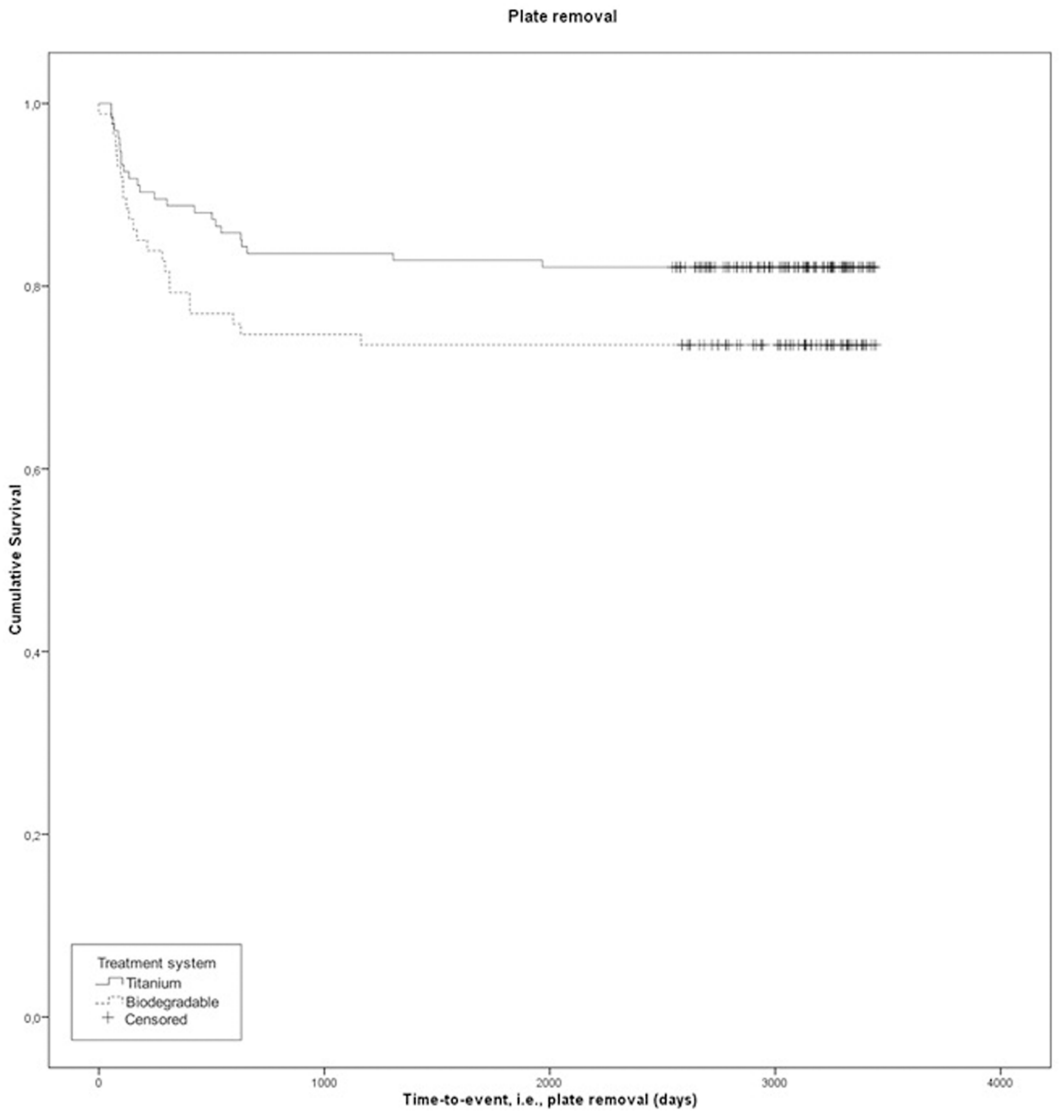
Kaplan-Meier curve of plate removal after long-term follow-up of all included patients (n = 221: titanium n = 134, biodegradable n = 87). Adjusted hazard ratio (HR) biodegradable = 2.0 (95% CI: 1.05–3.8), HR titanium = 1; p = 0.036.

The median (range) follow-up was 95 (77–111) and 98 (80–111) months in the titanium and biodegradable group, respectively (Table 3). Twenty two patients (16.4%) with a titanium system and 23 patients (26.4%) with a biodegradable system needed a second operation for plate/screws removal during the follow-up period (Table 3; Fig 2). Univariable plate removal analysis showed no significant difference between both groups (P = 0.070). However, in six of the 25 (24%) intra-operative switch patients, a second operation was needed to remove plates/screws. Therefore, the treatment variable, i.e. titanium or biodegradable, was analyzed using a Cox regression analysis, adjusting for ‘intra-operative switches’ (Fig 2; P = 0.036, hazard ratio (HR) biodegradable (95% CI) = 2.0 (1.05–3.8), HR titanium = 1). This states that the risk of necessity for biodegradable plate and screws removal is 2.0 times higher compared to titanium plates and screws after long-term follow-up.

**Table 3:** Outcome measures after long-term follow-up (i.e. >5 years post-operatively).

Description	Titanium (n)	Biodegradable (n)	P-value*
*Removal plate/screws (n (%))*^†^	22/134 (16.4%)	23/87 (26.4%)	0.036^‡^
*Removals surgical procedures*			0.318
*Removals osteotomies*	19/124 (15.3%)	23/79 (29.1%)	
BSSO	14/87 (16.1%)	19/55 (34.5%)	
Le Fort-I osteotomy	0/8	0/8	
Bi-maxillary osteotomy	5/29 (17.2%)	4/16 (25%)	
*Removals fractures*	3/10 (30.0%)	0/8	
Mandibular fracture	2/6 (33.3%)	0/4	
Le Fort-I fracture	0/1	0/0	
Zygoma fracture	1/3 (33.3%)	0/4	
*Self-evaluation of patient*^¶^			
Non-correct occlusion	14 (16.5%)	6 (10.7%)	0.461
Palpability plate/screws^#^	34 (41.5%)	4 (7.8%)	< 0.001
Swelling	3 (3.5%)	4 (7.1%)	0.436
Permanent	2 (2.4%)	3 (5.4%)	
Fluctuating	1 (1.2%)	1 (1.8%)	
Pain VAS (median (range))	0 (0–80)	0 (0–80)	0.736
MFIQ (median (range)) ^||^	18 (17–64)	17 (17–71)	0.110
Content with surgical procedure	81 (95.3%)	51 (91.1%)	0.483
*Follow-up time (median (range) in months)*^●^	95 (77–111)	98 (80–111)	0.458

*Tested two-tailed.

^†^Analyses performed on all included patients, without the Protocol violations and the Treatment Received violations (see Fig 1), n = 221: titanium n = 134, biodegradable n = 87).

^‡^ After adjusting for intra-operative switches. Univariable plate removal analysis showed no significant difference between both subgroups (P = 0.070).

^¶^ Analyses performed on all telephonically contacted patients after long-term follow-up (i.e. >5 years postoperatively), n = 141: titanium n = 85, biodegradable n = 56).

^#^The patients in whom the plates/screws were removed were not included in the analysis.

^||^The mandibular function was evaluated by the 17 questions of the MFIQ [1]; range 17–85; a higher score means worse function.

^●^ The follow-up of all telephonically contacted patients. The follow-up of all included patients was 98 (78–113) months.

Abbreviations: BSSO = bilateral-sagittal-split osteotomy, MFIQ = Mandibular Function Impairment Questionnaire, n = number, VAS = Visual Analogue Scale (range 0–100).

All 23 removals of the biodegradable group were due to clinical problems in the mandible and were only seen after an osteotomy. In the titanium group, 2 of the 22 removals (9.1%) were carried out in the mandible fracture patients. All other titanium removals, except one, were due to clinical problems after an osteotomy in the mandible. The main reason for plate/screws removal was abscess formation: 15 patients (65.2%) in the biodegradable group and 12 patients (50.1%) in the titanium group.

The titanium group showed significantly higher palpability of plate/screws (41.5%) compared to the biodegradable group (7.8%; P<0.001). No significant differences regarding occlusion, swelling, VAS pain scores, and MFIQ were found between both groups (Table 3). Additionally, no significant difference was found in terms of satisfaction of the performed surgical procedure. The main reason for dissatisfaction of the nine unsatisfied patients was insufficient occlusion (44.4%). Adjusting for the baseline characteristics (i.e. age, gender, and surgical procedure) did not significantly contribute to the other outcome variables (e.g. palpability, MFIQ, etc.; data not shown).

No significant differences were found between the intention-to-treat (ITT) and treatment received (TR) analysis. In addition, analysis showed no ‘center effect’ with regard to plate removal (data not shown).

## Discussion

This study showed that the biodegradable fixation system (Inion CPS) was removed significantly more often compared to the titanium fixation system (KLS Martin). The risk of necessity for biodegradable plate and screws removal was 2.0 times higher compared to titanium plates and screws, within a median follow-up of 99 months after surgery.

All biodegradable and nearly all titanium plate/screws removals were due to clinical problems related to the mandible. This could be due to the considerable forces acting on the plates/screws mounted to the mandible. This applies in particular to osteotomies of the mandible as there is no possibility of interfragmentary stability as could be the case in fractures of the mandible. Consequently, screws may loosen which could result in an inflammation. Additionally, it could be due to the morphology of the mandible and the lesser vascularization compared to other parts of the facial skeleton. There was no hardware removal in the 8 patients with fractures in the biodegradable group. Due to the small number of included fracture patients in this study, no firm conclusion regarding these surgical procedures can be drawn.

The main reason for plate and screws removal in both groups was abscess formation, which corresponds to the literature [20]. The causes of abscess formation are still unclear. During this trial, bacterial cultures in three patients with abscess formation in the biodegradable group showed a sterile inflammation [4]. A recent study reported similar results [21]. These inflammatory reactions could be a result of the degradation phase of biodegradable systems, which could trigger a foreign body reaction [8]. Additionally, during degradation, lactic acid is formed causing a low pH. This may contribute to an inflammatory reaction [22].

No significant differences were found in regard to occlusion, swelling, VAS pain scores, MFIQ, and satisfaction of the performed surgical procedure. Almost every contacted patient was free of pain and reported good mandibular function. The titanium group showed higher palpability of plate/screws compared to the biodegradable group. This is according to expectations as the vast majority of the biodegradable systems should have been dissolved in the human body. Nevertheless, it must be noted that in almost 8% of the contacted patients in the biodegradable group, the plate/screws were still palpable. In theory, this could still be the biodegradable fixation system. However, it could be that patients do not palpate plates or screws but rather the remodeled bone.

Occlusion was assessed by self-evaluation of the patient. Although the assessment of occlusion by patients themselves is subjective and may differ from assessment by a professional, we feel that the patient’s opinion regarding occlusion is of high importance. The discrepancy between the judgement of occlusion of a healthcare professional and the patient’s perception of their occlusion is therefore secondary.

Several observational studies have reported on plate removal in OMF surgery. Titanium systems are removed in 5–40% patients after trauma surgery [2,23] and in 7–27.5% patients after orthognathic surgery [1,24,25]. Biodegradable hardware is removed in 0–31% [10,26] and 0–3% of the patients [11,27,28], respectively. Randomized controlled trials that compare biodegradable with titanium fixation systems are scarce. One study showed 0% biodegradable and 31% titanium plate/screws removal after mandibular fractures [29]. Our study showed similar results, i.e. 0% biodegradable vs. 33.3% titanium plate removal in patients treated with mandibular fractures. A recent meta-analysis showed 7.9% (21/267) biodegradable vs. 5.4% (22/404) titanium plate removal after orthognathic surgery [5]. The follow-up periods of the included studies were 8 weeks [3], up to 1 year [30], and up to 2 years [4,31]. Discrepancies in follow-up period compared to our study may have led to different plate removal rates.

This study was a multicenter randomized controlled trial including four different hospitals. We found no center effect for plate removal. Therefore, it may be assumed that the results of this study could be applied to other hospitals using Inion CPS biodegradable and KLS Martin titanium systems.

Despite the RCT protocol declaring that asymptomatic plates would not be removed, 2 patients of the titanium group requested removal of asymptomatic plates/screws. We analyzed these patients as non-removals and censored them at the moment of plate removal in the survival analysis. Furthermore, 80 patients (36%) were lost to long-term follow-up. However, since our most important outcome measure was plate/screws removal, we could also evaluate the medical records of all included patients for this outcome measure. Theoretically, it is possible that patients who could not be contacted by telephone had their plate/screws removed in a hospital other than in which the patient’s primary surgery had been performed, though this is highly unlikely. A post-hoc power analysis showed that, to detect a difference of 5% less biodegradable plate removals with a power of 80% or 90%, 140 or 180 patients were needed in total, respectively. We included 221 patients in total, which, in theory, makes the present study overpowered. Finally, the intra-operative switches could have influenced plate removal rate. To minimalize this effect, we adjusted for these intra-operative switches in our survival analysis.

## Conclusion

In conclusion, regarding plate/screws removal after >5 years follow-up, the performance of the Inion CPS biodegradable system was inferior compared to the KLS Martin titanium system following fixation of mandibular, Le Fort-I, and zygomatic fractures, and bilateral sagittal split osteotomies (BSSO) and/or Le Fort-I osteotomies. However, due to the small number of included fracture patients in this study, no firm conclusion regarding these surgical procedures could be drawn. Finally, the intra-operative switches due to material failure of the biodegradable system [3] and the preferable cost-effectiveness of the titanium system [32] also argue against the usage of Inion CPS in the abovementioned surgical procedures.

## Supporting information

S1 CONSORT Checklist CONSORT Checklist 2010 checklist of information to include when reporting a randomised trial. (DOC)

S1 Protocol Protocol efficacy and safety aspects of biodegradable fixation systems: A randomized clinical trial. English version of the original trial protocol as approved by the local Medical Ethical Committees of the 4 participating hospitals in the Netherlands.
(DOC)

S2 Protocol Protocol effectiviteit En kosten aspecten van biodegradeerbare fixatie systemen: Een gerandomiseerde klinische studie. Dutch version of the original protocol as approved by the Medical Ethical Committees of the 4 participating hospitals in the Netherlands.
(DOC)

S1 Table Baseline characteristics and outcome measures of patients lost to long-term follow-up (i.e. >5 years) and patients not lost to long-term follow-up*. *Analyses performed on all included patients, without the Protocol violations and the Treatment Received violations (see Fig 1), n = 221. ^†^Tested two-tailed. Abbreviations: BSSO = bilateral-sagittal-split osteotomy, n = number.
(DOCX)
