# Supplementary material for: Comparison of the long-term clinical performance of a biodegradable and a titanium fixation system in maxillofacial surgery: A multicenter randomized controlled trial
Source: PLoS One. 2017 May 11;12(5):e0177152. doi: 10.1371/journal.pone.0177152 (PMC5426637; doi:10.1371/journal.pone.0177152)
Supplement: S1 Table — *Analyses performed on all included patients, without the Protocol violations and the Treatment Received violations (see Fig 1), n = 221. †Tested two-tailed. Abbreviations: BSSO = bilateral-sagittal-split osteotomy, n = number. (DOCX) [file pone.0177152.s004.docx]

**Table S1.** **Baseline characteristics and outcome measures of patients lost to long-term follow-up (i.e. >5 years) and patients not lost to long-term follow-up^*^**

| **Description** | **LTFU** | **Not LTFU** | **P-value**^†^ |
| --- | --- | --- | --- |
|  | **Baseline characteristics** | |  |
| *Surgical procedures* | 80 | 141 |  |
| BSSO | 48 (60%) | 94 (66.7%) | 0.402 |
| Le Fort-I osteotomy | 8 (10%) | 8 (5.7%) |  |
| Bi-maxillary osteotomy | 15 (18.8%) | 30 (21.3%) |  |
| Mandibular fracture | 4 (5.0%) | 6 (4.3%) |  |
| Le Fort-I fracture | 1 (1.3%) | 0 |  |
| Zygoma fracture | 4 (5.0%) | 3 (2.1%) |  |
| *Gender/age distribution* |  |  |  |
| Male | 33 (41.3%) | 65 (46.1%) | 0.573 |
| Female | 47 (58.8%) | 76 (53.9%) |  |
| Age (median (range) in years) | 25 (14-60) | 30 (15-59) | 0.050 |
|  | **Outcome measure** | |  |
| *Removal plate/screws (n (%))* | 19/80 (23.8%) | 26/141 (18.4%) | 0.386 |
